# Neurite Outgrowth-Promoting Compounds from Cockscomb Hydrolysate

**DOI:** 10.3390/nu14071422

**Published:** 2022-03-29

**Authors:** Takeru Koga, Akihiro Tai

**Affiliations:** 1 Graduate School of Advanced Technology and Science, Tokushima University, 2-1 Minamijosanjima-cho, Tokushima 770-8506, Japan; c502044005@tokushima-u.ac.jp; 2 Graduate School of Technology, Industrial and Social Science, Tokushima University, 2-1 Minamijosanjima-cho, Tokushima 770-8513, Japan

**Keywords:** cockscomb hydrolysate, amino acid, PC12 cells, NGF, dibutyryl cyclic AMP

## Abstract

Cockscomb hydrolysate was found to have neurite outgrowth-promoting activity in PC12 cells. To investigate the neurite outgrowth-promoting compounds derived from cockscomb hydrolysate, bioassay-guided purification was carried out. Purified active fractions were obtained by liquid–liquid partition, followed by column chromatography. High-performance liquid chromatography and proton nuclear magnetic resonance analyses of the purified active fractions clarified that the main compounds are threonine, alanine, valine, and methionine. By screening for 20 kinds of amino acids, it was shown that valine and methionine, but not threonine and alanine, have neurite outgrowth-promoting activity. The results of activity evaluation of the mixture of amino acids indicated that alanine enhanced the activity of valine and that the mixture of valine and methionine showed a higher ratio of neurite formation than did each of them alone. On the other hand, dipeptides formed by valine and methionine showed weak neurite outgrowth-promoting activity. A mixture of threonine, alanine, valine, and methionine at the same concentrations as those in cockscomb hydrolysate showed neurite outgrowth-promoting activity comparable to that of cockscomb hydrolysate although threonine, alanine, valine, and methionine alone did not show activity at their concentrations in cockscomb hydrolysate. Therefore, the strong neurite outgrowth-promoting activity of cockscomb hydrolysate was considered to be due to the synergistic effect of threonine, alanine, valine, and methionine.

## 1. Introduction

Foods have not only the function of providing nutrients to the living body and providing taste preferences sensuously, but also the function of regulating various systems of the body through components in the food [1]. It is believed that the regulatory functions of body systems provided by foods can help maintain and improve health and assist in the prevention of and recovery from lifestyle-related diseases. In recent years, products that emphasize the functionality of food as health foods have become widely accepted due to the increase in awareness of health with the increase in average life expectancy. Recently, there has been widespread use of products that improve absorption and functionality by enzymatic or chemical hydrolysis. It has been reported that mixtures of chitosan oligosaccharides, consisting mainly of disaccharides to octasaccharides, obtained by hydrolysis of chitosan, show growth-promoting effects on bifidobacteria and lactobacilli [2]. Those findings indicate that chitosan oligosaccharides have excellent prebiotic effects. It has also been reported that mixtures of disaccharides to octasaccharides derived from chitosan increased the activity of lysozyme in serum up to two times and improved the biological defense function when the chitosan oligosaccharides were administered to rabbits [3]. Chondroitin sulfate oligosaccharides hydrolyzed chemically inhibit IgE-mediated allergic reactions by down-regulation of the Th2 response in mice [4]. Sardine peptide, obtained by protease degradation of protein derived from sardines, inhibits the action of the angiotensin-converting enzyme and helps to maintain normal blood pressure [5,6]. Albumen hydrolysate has been shown to lower plasma cholesterol and triglyceride levels [7]. Ingestion of pork peptides hydrolyzed with papain, a plant protease extracted from papaya fruit, decrease serum cholesterol and increase the fecal excretion of cholesterol and bile acids [8].

Cockscomb (chicken’s comb or rooster comb), is a food that is rich in hyaluronic acid and collagen protein. Hyaluronic acid has been shown to promote wound healing [9] and mitigate DNA damage caused by reactive oxygen species in human conjunctival epithelial cells [10]. Tetrasaccharides obtained by chemically or enzymatically hydrolyzing hyaluronic acid have been reported to promote neuron regeneration in vivo [11]. Ingestion of collagen peptide suppresses UV-B-induced decreases in skin hydration, hyperplasia of the epidermis, and decreases in soluble type I collagen [12]. In recent years, cockscomb hydrolyzed by some proteases has been reported to improve knee joint pain [13], but the agents have not been clarified. There has been little research on cockscomb hydrolysate, and it may have unreported effects. A preliminary study has shown that cockscomb hydrolysate promotes neurite outgrowth in PC12 cells (unpublished data). Therefore, in this study, the neurite outgrowth-promoting compounds in cockscomb hydrolysate were purified and their activities were investigated.

## 2. Materials and Methods

### 2.1. Materials

Cockscomb hydrolysate was provided by Laimu Co., Ltd. (Yokohama, Japan). Methanol, ethanol, acetonitrile, acetone, ethyl acetate (EtOAc), 10 × D-PBS(-), Giemsa stain solution, 25% glutaraldehyde solution, L-cysteine hydrochloride monohydrate, glycine, L-histidine, L(-)-valine, L-tyrosine, L(-)-threonine, D(-)-valine, and D-methionine were obtained from FUJIFILM Wako Pure Chemical Corporation (Osaka, Japan). L-Methionine, L-leucine, L(-)-phenylalanine, L(+)-lysine monohydrochloride, L-glutamic acid, L-aspartic acid, L(+)-glutamine, and L-arginine were purchased from Junsei Chemical Co., Ltd. (Tokyo, Japan). L-Serine and L-isoleucine were obtained from Peptide Institute, Inc. (Osaka, Japan). H-Val-Met-OH and H-Met-Val-OH were purchased from Kokusan Chemical. Co., Ltd. (Tokyo, Japan). RPMI-1640 medium and dibutyryl cyclic AMP (Bt_2_cAMP) were purchased from Sigma-Aldrich Japan (Tokyo, Japan). Penicillin–streptomycin mixed solution, sodium dihydrogenphosphate dehydrate, disodium hydrogenphosphate, L(-)-proline, L-asparagine monohydrate, L-tryptophan, and L-α-alanine were obtained from Nacalai Tesque (Kyoto, Japan). Fetal bovine serum (FBS) (Lot. 42F9155K) and horse serum (HS) (Lot. 1517707) were obtained from Gibco (Waltham, MA, USA). Cell-matrix type I–P (collagen) was purchased from Nitta Gelatin (Osaka, Japan). Ninety-six-well plates (167008, Nunc) were obtained from Thermo Fisher Scientific K.K. (Tokyo, Japan). Recombinant rat β-nerve growth factor protein (NGF) (Cat. 556-NG) was purchased from R&D SYSTEMS (USA). Diaion HP20 (Mitsubishi Chemical Corporation, Tokyo, Japan), Wakogel 50NH_2_ (FUJIFILM Wako Pure Chemical Corporation), TOYOPEARL HW-40F (Tosoh Corporation, Tokyo, Japan), and Chromatorex ARG (Fuji Silysia Chemical Ltd., Kasugai, Japan) were used for column chromatography.

### 2.2. Purification of Fractions Showing Neurite Outgrowth-Promoting Activity from Cockscomb Hydrolysate

Cockscomb hydrolysate (97.3 g/1970 mL) was added to 7880 mL of ethanol (with the final concentration of ethanol being 80%) and the mixture stood for 24 h at 4 °C. After 24 h, the cockscomb hydrolysate containing ethanol was centrifuged at 3773× *g* for 10 min at 4 °C and separated into the supernatant and precipitate. The supernatant showing neurite outgrowth-promoting activity was concentrated to an approximate volume of 760 mL. The concentrated supernatant was partitioned with EtOAc (760 mL, 3 times) to obtain a water layer (58.3 g, dry wt.) and an EtOAc layer (408.3 mg, dry wt.). The water layer, which showed neurite outgrowth-promoting activity (Figure S2 in the Supplementary Materials), was applied to a Diaion HP20 column (12.5 cm i.d. × 35.0 cm) and eluted with a stepwise H_2_O-MeOH gradient (10/0, 8/2, 6/4, 4/6, and 2/8 each 4 L, *v*/*v*), and fractions eluted with 100% H_2_O showed activity. The active fraction was further applied to a Diaion HP20 column (5.0 cm i.d. × 40.5 cm) and eluted with 2.4 L of H_2_O to obtain active fraction A and fraction B. Fraction A was chromatographed on Wakogel 50NH_2_ and eluted with a stepwise acetone-H_2_O gradient (8/2, 7.5/2.5, 7/3, and 6/4 each 345 mL, 4/6: 690 mL, *v*/*v*) to obtain active fraction C eluted with 80% acetone, and fraction D eluted with 75% acetone. Fraction C was purified by TOYOPEARL HW-40F (1.5 cm i.d. × 110.0 cm) with 380 mL of H_2_O to obtain active fraction E (24.2 mg). On the other hand, fraction D was purified by TOYOPEARL HW-40F (1.5 cm i.d. × 110.0 cm) with 400 mL of H_2_O to obtain active fraction F (7.9 mg) and fraction G (8.1 mg). Fraction B was applied to a TOYOPEARL HW-40C (2.5 cm i.d. × 81.0 cm) with 1.0 L of H_2_O to obtain an active fraction. The active fraction was purified by TOYOPEARL HW-40F (1.5 cm i.d. × 112.0 cm) with 300 mL of H_2_O and Chromatorex ARG (1.5 cm i.d. × 5.7 cm) with a stepwise acetonitrile-H_2_O gradient (10/0, 9.5/0.5, 9/1, 8/2, 7/3, 6/4, and 5/5, *v*/*v*) in order to obtain an active fraction eluted with 70% aqueous acetonitrile solution. The obtained fraction was purified by Chromatorex ARG (1.0 cm i.d. × 26.5 cm) with acetonitrile-H_2_O (7/3, *v*/*v*) 3 times to obtain active fraction H (1.3 mg). As a result of ^1^H-nuclear magnetic resonance (NMR) analyses and high-performance liquid chromatography (HPLC) co-chromatography analyses of the four active fractions E, F, G, and H, it was clarified that the main components of each fraction were threonine, alanine, valine, and methionine.

### 2.3. Neurite Outgrowth-Promoting Activity

Neurite outgrowth-promoting activity was evaluated by modifying the method previously reported in our laboratory [14,15]. PC12 cells were purchased from RIKEN BRC Cell Bank (Tsukuba, Japan). The cells were grown in RPMI-1640 supplemented with 10% HS, 5% FBS, 100 U/mL penicillin G, and 100 μg/mL streptomycin (basal medium) at 37 °C in a humidified atmosphere of 95% air/5% CO_2_. The basal medium was changed every one or two days. Neurite outgrowth-promoting activity was evaluated using PC12 undifferentiated cells at passage numbers 8–15. The purified fractions of cockscomb hydrolysate were tested for evaluation of the activity as samples. In addition, amino acids having the activity were explored by screening for 20 kinds of L-amino acids. The activities of dipeptides composed of valine and methionine (H-Val-Met-OH and H-Met-Val-OH) were also evaluated. The evaluated amino acids and dipeptides were commercially available standards. PC12 cells from stock culture were suspended in the medium and plated at 4.0 × 10^3^ cells/90 μL/well (for evaluation in the presence of Bt_2_cAMP) or 2.0 × 10^3^ cells/90 μL/well (for evaluation in the presence of NGF) in 96-well-plates coated with collagen and incubated in a humidified atmosphere of 5% CO_2_ at 37 °C. After 24 h, 5 μL of Bt_2_cAMP at 10 mM (final concentration: 0.5 mM) or NGF at 200 ng/mL (final concentration: 10 ng/mL) and 5 μL of each sample or the control (medium only) were added to the culture medium. (The final concentration of each sample is indicated in the figures) At 24 h after the addition of Bt_2_cAMP and samples, or at 48 h after the addition of NGF and samples, the medium was aspirated, and PC12 cells were fixed with phosphate buffer (pH 7.2, 100 mM) containing 1% glutaraldehyde and stained by Giemsa stain solution. Then the 96-well-plates were washed twice with Milli-Q grade water. The number of cells bearing neurites longer than twice the diameter of one cell body after treatment was divided by the total number of cells, which was 300–400 cells per well.

## 3. Results and Discussion

### 3.1. Purification of Neurite Outgrowth-Promoting Compounds from Cockscomb Hydrolysate

Nerve cells depend on nerve growth factor (NGF) to maintain their survival and differentiation [16]. NGF elongates neurites in PC12 cells, which have been widely used as a model for neural differentiation [17]. Cockscomb hydrolysate promoted neurite formation in the presence of NGF but did not elongate neurites in the absence of NGF (Figure S1 in the Supplementary Materials). These results indicated that cockscomb hydrolysate has neurite outgrowth-promoting activity but not NGF-mimicking activity. To investigate neurite outgrowth-promoting compounds from cockscomb hydrolysate, bioassay-guided purification was carried out. Dibutyryl cyclic AMP (Bt_2_cAMP) is known as a substance that induces neurite formation similar to NGF. NGF binds to the tropomyosin receptor kinase A receptor and extends neurites via a signal-regulated kinase (ERK) [18,19]. As another signaling pathway, NGF increases intracellular cAMP levels and induces neurite formation [20]. Bt_2_cAMP, a membrane-permeable cAMP derivative, is metabolized to cAMP in cells and exhibits neurite outgrowth activity. Therefore, in order to efficiently evaluate neurite outgrowth-promoting activity in a short time and to purify the active compounds, Bt_2_cAMP was applied as a neurite-formation-inducer in PC12 cells.

**Figure 1:**
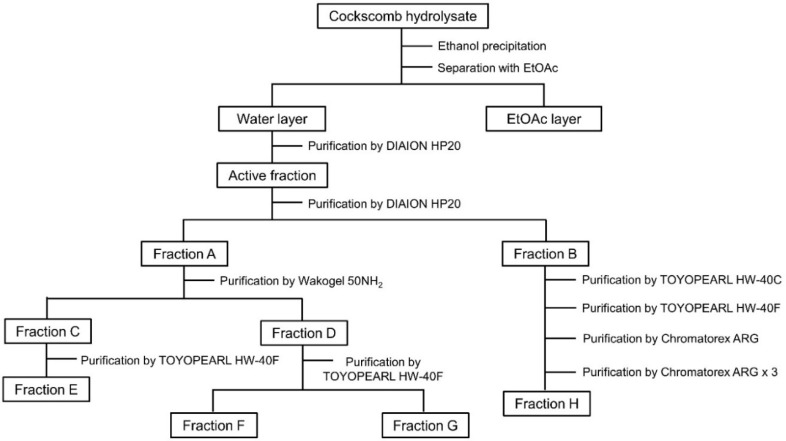
Purification scheme for neurite outgrowth-promoting compounds contained in cockscomb hydrolysate.

Active fractions from cockscomb hydrolysate were purified by the process shown in Figure 1. Cockscomb hydrolysate was precipitated with 80% ethanol. After centrifugation, the active supernatant was concentrated and then partitioned with EtOAc. Both the water layer and the EtOAc layer showed activity in the presence of Bt_2_cAMP in PC12 cells (Figure S2 in the Supplementary Materials). Since the dry weight of the water layer was overwhelmingly larger than that of the EtOAc layer, the water layer was further purified by column chromatographies. Then the active water layer was chromatographed on a Diaion HP20 column twice to obtain active fractions A and B. Fraction A was purified by Wakogel 50NH_2_ to obtain fractions C and D, showing activity. Fraction C was purified with TOYOPEARL HW-40F to obtain active fraction E (24.2 mg). On the other hand, fraction D was purified by TOYOPEARL HW-40F to obtain fraction F (7.9 mg) and fraction G (8.1 mg). Fraction B was purified by TOYOPEARL HW-40C, TOYOPEARL HW-40F and Chromatorex ARG, in that order. Furthermore, the purified fraction was chromatographed on Chromatorex ARG three times to obtain active fraction H (1.3 mg). The neurite outgrowth-promoting activities of the active fractions E-H are shown in Figure S3 in the Supplementary Materials. Some active fractions from cockscomb hydrolysate showed a decline in, or loss of, activity as the purification proceeded. Hence, the complete isolation of active compounds was abandoned, and active fractions containing small amounts of impurities were subjected to structural analyses. The main compounds of fractions E, F, G, and H were identified as threonine, alanine, valine, and methionine, respectively, on the basis of ^1^H-NMR analyses and HPLC co-chromatography analyses with available authentic standards (Figures S4–S11 in the Supplementary Materials). It was clarified that an amino acid is the main component of each of the four neurite outgrowth-promoting fractions obtained by purification from cockscomb hydrolysate.

### 3.2. Screening for Amino Acids Having Neurite Outgrowth-Promoting Activity

**Figure 2:**
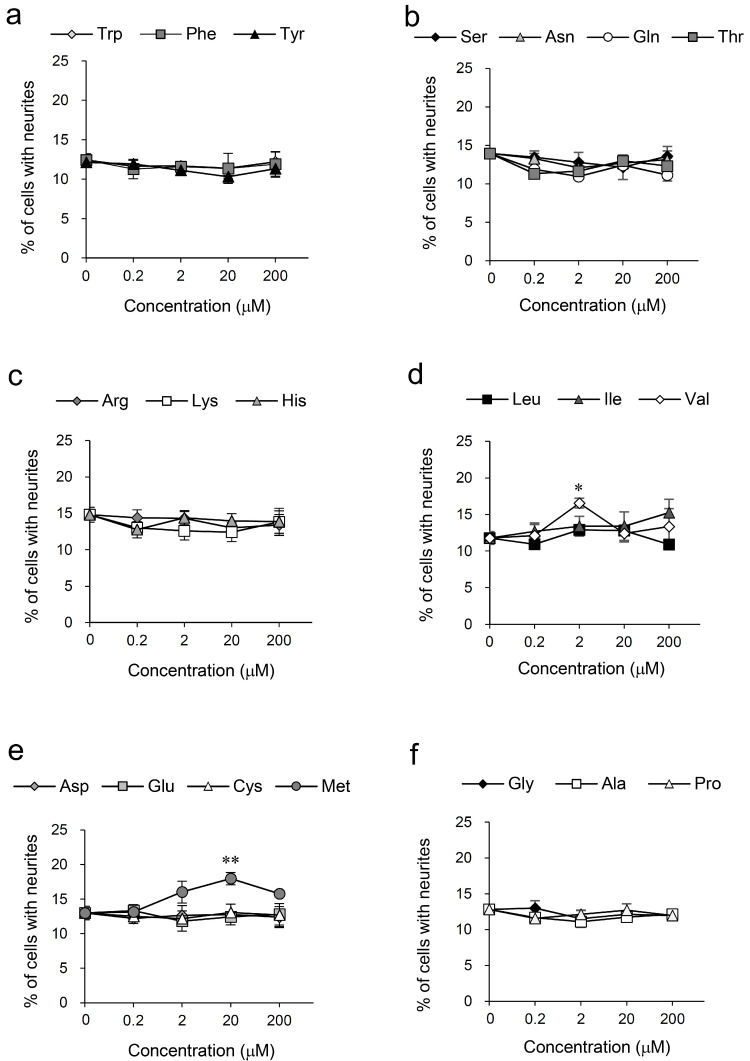
Neurite outgrowth-promoting activities of 20 kinds of amino acids in the presence of Bt_2_cAMP in PC12 cells. Promoting activities of Trp, Phe, and Tyr (**a**), Ser, Asn, Gln, and Thr (**b**), Arg, Lys, and His (**c**), Leu, Ile, and Val (**d**), Asp, Glu, Cys, and Met (**e**), and Gly, Ala, and Pro (**f**) for neurite formation induced by Bt_2_cAMP in PC12 cells. PC12 cells were plated at 4.0 × 10^3^ cells/well and cultured with the amino acids at 0.2–200 μM in the presence of 0.5 mM of Bt_2_cAMP. The extent of neurite outgrowth was measured at 24 h and is expressed as the mean percentage of 300–400 cells. The data represent means ± standard deviation (SD) from three independent experiments. * *p* < 0.05, ** *p* < 0.01 (Dunnett’s test), as compared with the control (0.5 mM Bt_2_cAMP only).

Activity-guided purification of cockscomb hydrolysate yielded four fractions, mainly composed of threonine, alanine, valine, and methionine, respectively, that showed neurite outgrowth-promoting activity in the presence of Bt_2_cAMP in PC12 cells. Amino acids having the activity were explored by screening for 20 kinds of L-amino acids. Among the 20 amino acids, valine and methionine were discovered as substances showing neurite outgrowth-promoting activity (Figure 2d,e). Valine and methionine were consistent with the main components of active fractions G and H. The activities of the L and D forms of valine and methionine were almost the same (Figure S12 in the Supplementary Materials). The results indicate that there was no effect of the activity between their optical isomers. Other L-amino acids did not show neurite outgrowth-promoting activity (Figure 2). Threonine and alanine, the main components of active fractions E and F, did not show activity (Figure 2b,f), suggesting that other components contained in small amounts in each fraction are active compounds.

**Figure 3:**
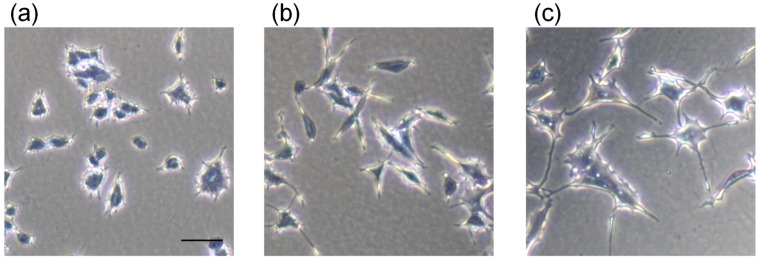
Photomicrographs of PC12 cells treated with medium only (**a**), 0.5 mM Bt_2_cAMP (**b**), and mixture of 2 μM valine and 0.5 mM Bt_2_cAMP (**c**). PC12 cells were plated at 4.0 × 10^3^ cells/well and cultured with medium only, Bt_2_cAMP at 0.5 mM, or mixture of valine at 2 μM and Bt_2_cAMP at 0.5 mM. After 24 h, treated cells were fixed with 1% glutaraldehyde and stained with Giemsa stain solution. Scale bar = 50 μm.

The morphological appearance of PC12 cells treated with valine, which showed the strongest neurite outgrowth-promoting activity as a result of screening, is shown in Figure 3. Valine is an essential amino acid and a type of branched-chain amino acid (BCAA). BCAAs have been shown to inhibit muscle wasting due to exercise [21], improve anorexia and nutritional status [22], and improve symptoms in patients with hepatic encephalopathy [23]. It has also been reported that the concentration of valine in the serum of Alzheimer disease patients was decreased compared to that in healthy subjects, but the relationship between valine and Alzheimer disease has not been clarified [24]. Methionine is an essential amino acid and one of the sulfur-containing amino acids. Methionine is known to have hepatoprotective effects [25] and to inhibit decreased immunocompetence in HIV-infected patients [26]. It has also been reported that S-adenosylmethionine may play a neuroprotective role by increasing the expression of endogenous brain-derived neurotrophic factor [27]. As described above, valine and methionine have various functions, and some functions that may be beneficial for Alzheimer disease have been reported. However, studies related to the neurite-formation of valine and methionine have not been reported.

### 3.3. Neurite Outgrowth-Promoting Activities of Mixtures of Amino Acids

**Figure 4:**
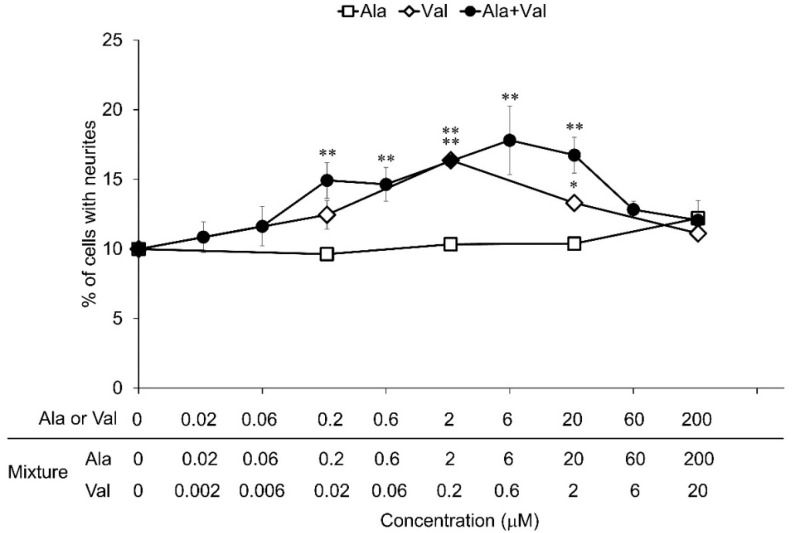
Neurite outgrowth-promoting activities of alanine, valine, and their mixture in the presence of Bt_2_cAMP in PC12 cells. PC12 cells were plated at 4.0 × 10^3^ cells/well and cultured with the samples (final concentrations: from 0.02 μM of alanine and 0.002 μM of valine to 200 μM of alanine and 20 μM of valine) in the presence of Bt_2_cAMP (0.5 mM). The extent of neurite outgrowth was measured at 24 h and is expressed as the mean percentage of 300–400 cells. The data represent means ± standard deviation (SD) from three independent experiments. * *p* < 0.05, ** *p* < 0.01 (Dunnett’s test), as compared with the control (0.5 mM Bt_2_cAMP only).

Valine and methionine, which were the main components of active fractions G and H, respectively, showed significant neurite outgrowth-promoting activity, while threonine and alanine, which were the main components of active fractions E and F, respectively, did not show activity on screening of amino acids. It was thought that fractions E and F exerted their activities due to the effect of compounds mixed in a small amount. To clarify the mixed compounds in fractions E and F, ^1^H-NMR and HPLC analyses of their fractions were performed. ^1^H-NMR and HPLC analyses showed that fraction F contained a mixture of alanine and valine in a ratio of approximately 10:1 (Figures S6 and S7 in the Supplementary Materials). However, in fraction E, a small amount of mixed compounds could not be identified by these analyses. In order to investigate the neurite outgrowth-promoting activity of the mixture of alanine and valine contained in fraction F, the activity of the mixture of alanine and valine in a ratio of 10:1 was evaluated and compared with the activities of alanine and valine (Figure 4). Mixtures of alanine and valine in a ratio of 10:1 showed significant activities in the concentration range from 0.2 μM of alanine and 0.02 μM of valine to 20 μM of alanine and 2 μM of valine. Alanine alone did not show activity, and valine alone showed significant activity at concentrations of 2 μM to 20 μM. The minimum concentration of valine in the mixture required to exert activity was 0.02 μM, whereas valine alone required a concentration of 2 μM to exert activity. The ratios of neurite outgrowth induced by the mixtures were the same or higher than that induced by valine only. These results indicate that alanine enhances the activity of valine under the condition of co-existence with valine. In addition, it was predicted that amino acids may exhibit even stronger neurite outgrowth-promoting activity in their mixtures.

**Figure 5:**
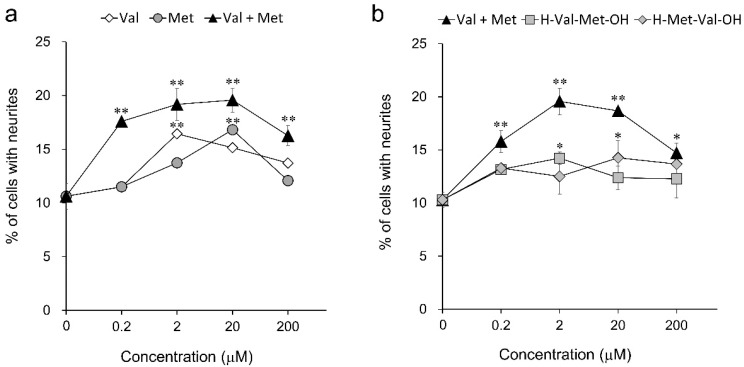
Neurite outgrowth-promoting activities of valine, methionine, their mixture, and their dipeptide in the presence of Bt_2_cAMP in PC12 cells. (**a**) Promoting activities of valine and methionine and a mixture of equal amounts of valine and methionine for neurite formation induced by Bt_2_cAMP in PC12 cells. (**b**) Promoting activities of a mixture of equal amounts of valine and methionine and their dipeptides (H-Val-Met-OH, H-Met-Val-OH) for neurite formation induced by Bt_2_cAMP in PC12 cells. PC12 cells were plated at 4.0 × 10^3^ cells/well and cultured with the samples at concentrations of 0.2–200 μM in the presence of 0.5 mM of Bt_2_cAMP. The extent of neurite outgrowth was measured at 24 h and is expressed as the mean percentage of 300–400 cells. The data represent means ± standard deviation (SD) from three independent experiments. * *p* < 0.05, ** *p* < 0.01 (Dunnett’s test), as compared with the control (0.5 mM Bt_2_cAMP only).

Valine and methionine were found to show neurite outgrowth-promoting activities by screening for amino acids (Figure 2). Since it was assumed that the activity was enhanced by mixing valine and methionine based on the results for the activity of a mixture of alanine and valine, the neurite outgrowth-promoting activity of a mixture of valine and methionine in equal amounts was investigated. The activities of dipeptides composed of valine and methionine (H-Val-Met-OH and H-Met-Val-OH) were also evaluated. The mixtures of valine and methionine showed significant activity in a wide range of concentrations from 0.2 μM to 200 μM, and valine and methionine had the highest activity at concentrations of 2 μM and 20 μM, respectively (Figure 5a). On the other hand, dipeptides composed of valine and methionine showed weak neurite outgrowth-promoting activity (Figure 5b). The mixture of valine and methionine showed sufficiently strong activity at concentrations lower than those at which valine and methionine alone showed activity. In addition, the neurite formation ratio of the mixture was higher than that of valine and methionine alone. The mixture of valine and methionine also showed concentration-dependent activity in the presence of NGF, and the neurite-formation ratio of the mixture was higher than that of valine and methionine, respectively (Figure S13 in the Supplementary Materials). The dipeptides did not show activity in the presence of NGF. These results suggested that the mixture of valine and methionine has a synergistic promoting effect on neurite outgrowth.

**Figure 6:**
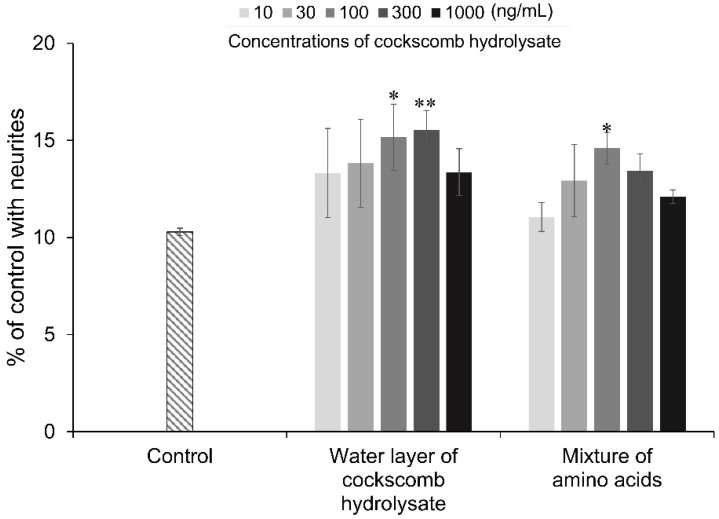
Neurite outgrowth-promoting activities of the water layer of cockscomb hydrolysate and a mixture of threonine, alanine, valine, and methionine in the presence of Bt_2_cAMP. PC12 cells were plated at 4.0 × 10^3^ cells/well and cultured with 10–1000 ng/mL of the water layer or mixture of amino acids consists of threonine, alanine, valine, and methionine corresponding to the concentration of cockscomb hydrolysate. The concentration of each amino acid in the mixture corresponding to the water layer of cockscomb hydrolysate at 100 ng/mL is 6.88 ng/mL for threonine, 2.31 ng/mL for alanine, 1.74 ng/mL for valine, and 0.97 ng/mL for methionine. The extent of neurite outgrowth was measured at 24 h and is expressed as the mean percentage of 300–400 cells. The data represent means ± standard deviation (SD) from three independent experiments. * *p* < 0.05, ** *p* < 0.01 (Dunnett’s test), as compared with the control (0.5 mM Bt_2_cAMP only).

Finally, the neurite outgrowth-promoting activity of a mixture of threonine, alanine, valine, and methionine, which are the main components of the four active fractions purified from cockscomb hydrolysate, was investigated. HPLC analyses revealed that 100 ng/mL of the water layer from cockscomb hydrolysate contained 6.88 ng/mL of threonine, 2.31 ng/mL of alanine, 1.74 ng/mL of valine, and 0.97 ng/mL of methionine (Figures S14 and S15, and Table S1 in the Supplementary Materials). Threonine, alanine, valine, and methionine were mixed in the ratio, and neurite outgrowth-promoting activity of the mixture was evaluated at the same concentrations as those in the water layer. The mixture of threonine, alanine, valine, and methionine showed neurite outgrowth-promoting activity at concentrations equivalent to those in 100 ng/mL of the water layer, that is, 6.88 ng/mL of threonine, 2.31 ng/mL of alanine, 1.74 ng/mL of valine, and 0.97 ng/mL of methionine (Figure 6). The water layer showed significant activity at 100 ng/mL and 300 ng/mL. The profile of the neurite outgrowth-promoting activity at each concentration of the mixture and the water layer was quite similar. Valine and methionine required at least 2 μM and 20 μM, that is, 234.3 ng/mL and 2.98 μg/mL, respectively, to show activity (Figure 2). Valine and methionine alone required much higher concentrations than those contained in the water layer of the cockscomb hydrolysate to exert neurite outgrowth-promoting activity. Threonine and alanine alone did not show activity. Therefore, the strong neurite outgrowth-promoting activity of cockscomb hydrolysate was considered to be due to the synergistic effect of threonine, alanine, valine, and methionine.

Recently, the number of patients with dementia caused by Alzheimer disease has been increasing [28]. A decrease in NGF is associated with one of the causes of Alzheimer disease. With aging, the level of NGF in the hippocampus, which is associated with memory and learning, decreases [29], and the inability to maintain neurite formation is thought to lead to neurite damage and neuronal death, resulting in a decline in cognitive function [30]. Since it has been reported that the administration of NGF to old rats with low levels of learning ability for one month improved learning ability [31], it was thought that increasing the amount of NGF in the brain would improve Alzheimer disease. However, application of NGF as a medicine for Alzheimer disease is difficult because NGF cannot pass through the blood–brain barrier due to its physicochemical properties [32,33]. Hence, compounds that show an NGF-enhancing effect and can cross the blood–brain barrier are required for preventing Alzheimer disease. In the present study, a novel activity of amino acids was found to be neurite outgrowth-promoting activity, which is one of the NGF-enhancing effects. Amino acids can cross the blood–brain barrier and reach the brain [34]. These facts provide evidence that cockscomb hydrolysate may be an effective food for the prevention of Alzheimer disease.

## 4. Conclusions

Four fractions showing significant neurite outgrowth-promoting activity at a low concentration in the presence of Bt_2_cAMP in PC12 cells were purified from cockscomb hydrolysate. It was clarified by ^1^H-NMR and HPLC analyses that the main components of the active fractions were threonine, alanine, valine, and methionine. In screening for amino acids, commercially available standards, valine and methionine had activity, but threonine and alanine did not have activity. ^1^H-NMR and HPLC analyses of the active fraction, which was mainly composed of alanine, indicated that valine was mixed in the fraction at a ratio of approximately one-tenth. On the other hand, the compounds mixed in small amounts in the other three active fractions were not identified. The mixture of alanine (not having activity) and valine (having activity) showed a higher ratio of neurite outgrowth over a wider range of concentrations than that of valine alone, suggesting that alanine enhances the activity of valine. The mixture of valine and methionine, which each have activity, showed a higher neurite outgrowth-promoting ratio than that of either valine or methionine alone over a wide range of concentrations. Furthermore, the activity of a mixture consisting of threonine, alanine, valine, and methionine at concentrations corresponding to those in the water layer from cockscomb hydrolysate was very similar to that of the water layer. None of the amino acids showed activity alone at the concentrations contained in the mixture that did show significant activity. The results suggest that the strong neurite outgrowth-promoting activity of cockscomb hydrolysate is due to the synergistic effect of several kinds of amino acids.

## Data Availability

Not applicable.

## Supplementary Materials

The following supporting information can be downloaded at https://www.mdpi.com/article/10.3390/nu14071422/s1; Figure S1: Neurite outgrowth activity of cockscomb hydrolysate in the presence or in the absence of NGF; Figure S2: Neurite outgrowth-promoting activities of cockscomb hydrolysate, supernatant, and precipitate by ethanol precipitation, water layer, and EtOAc layer obtained by liquid separation of the supernatant in the presence of Bt_2_cAMP; Figure S3: Neurite outgrowth-promoting activities of fractions E-H in the presence of Bt_2_cAMP; Figure S4: ^1^H-NMR spectrum of fraction E; Figure S5: HPLC analyses of fraction E; Figure S6: ^1^H-NMR spectrum of fraction F; Figure S7: HPLC analyses of fraction F; Figure S8: ^1^H-NMR spectrum of fraction G; Figure S9: HPLC analyses of fraction G; Figure S10: ^1^H-NMR spectrum of fraction H; Figure S11: HPLC analyses of fraction H; Figure S12: Neurite outgrowth-promoting activities of L-valine, D-valine, L-methionine, and D-methionine in the presence of Bt_2_cAMP in PC12 cells; Figure S13: Neurite outgrowth-promoting activities of valine, methionine, their mixture, and their dipeptides in the presence of NGF in PC12 cells; Figure S14: Calibration curves of the reference standards of threonine, alanine, valine, and methionine; Figure S15: HPLC analysis of the water layer of cockscomb hydrolysate; Table S1: Quantification of threonine, alanine, valine, and methionine in the water layer (5 μg/mL) of cockscomb hydrolysate.
