# Supplementary material for: Neurite Outgrowth-Promoting Compounds from Cockscomb Hydrolysate
Source: Nutrients. 2022 Mar 29;14(7):1422. doi: 10.3390/nu14071422 (PMC9002945; doi:10.3390/nu14071422)
Supplement: Supplementary file 1 [file nutrients-14-01422-s001.zip › nutrients-1609343-supplementary.pdf]

Supplementary Materials

Neurite outgrowth-promoting compounds from cockscomb hydrolysate

Takeru Koga[1] and Akihiro Tai[2,*]

[1] *Graduate School of Advanced Technology and Science, Tokushima University, 2-1 Minamijosanjima-cho, Tokushima 770-8506, Japan*

[2] *Graduate School of Technology, Industrial and Social Sciences, Tokushima University, 2-1 Minamijosanjima-cho, Tokushima 770-8513, Japan*

*** Corresponding author**

E-mail address: atai@tokushima-u.ac.jp

Contents:

Materials and methods

Figure S1. Neurite outgrowth activity of cockscomb hydrolysate in the presence or in the absence of NGF.

Figure S2. Neurite outgrowth-promoting activities of cockscomb hydrolysate, supernatant, and precipitate by ethanol precipitation, water layer, and EtOAc layer obtained by liquid separation of the supernatant in the presence of Bt$_2$cAMP.

Figure S3. Neurite outgrowth-promoting activities of fractions E-H in the presence of Bt$_2$cAMP.

Figure S4. ^{1}H-NMR spectrum of fraction E.

Figure S5. HPLC analyses of fraction E.

Figure S6. ^{1}H-NMR spectrum of fraction F.

Figure S7. HPLC analyses of fraction F.

Figure S8. ^{1}H-NMR spectrum of fraction G.

Figure S9. HPLC analyses of fraction G.

Figure S10. ^{1}H-NMR spectrum of fraction H.

Figure S11. HPLC analyses of fraction H.

Figure S12. Neurite outgrowth-promoting activities of L-valine, D-valine, L-methionine, and D-methionine in the presence of Bt$_2$cAMP in PC12 cells.

Figure S13. Neurite outgrowth-promoting activities of valine, methionine, their mixture, and their dipeptides in the presence of NGF in PC12 cells.

Figure S14. Calibration curves of the reference standards of threonine, alanine, valine, and methionine.

Figure S15. HPLC analysis of the water layer of cockscomb hydrolysate.

Table S1. Quantification of threonine, alanine, valine, and methionine in the water layer (5 μg/mL) of cockscomb hydrolysate.

Materials and methods

Materials

Methanol, *o*-phthalaldehyde (OPA), sodium acetate, boric acid, and sodium hydroxide were obtained from FUJIFILM Wako Pure Chemical Corporation (Osaka, Japan). Brij 35P was purchased from Sigma-Aldrich Japan (Tokyo, Japan). Deuterium oxide, 2-mercaptoethanol were obtained from Nacalai Tesque (Kyoto, Japan).

Measurements of ^{1}H-NMR spectra

^{1}H-NMR spectra were obtained on a JEOL NMR system (ECZ400) 400 MHz instrument.

Method of high performance liquid chromatography analyses

High performance liquid chromatography (HPLC) was performed by using a liquid chromatograph pump (Shimadzu Corporation, Kyoto, Japan), a fluorescence detector RF-10AXL (Shimadzu Corporation), a column oven CTO-10A (Shimadzu Corporation), a degasser DGU-14A (Shimadzu Corporation), and an integrator ADL-2020 (LC Science, Nara, Japan). Amino acids or cockscomb hydrolysate incubated with OPA reagent which is containing 1.55 g of boric acid, 0.7 g of sodium hydroxide, 0.05 g Brij 35P, 0.1 mL of 2-mercaptoethanol, and 0.04 g of OPA for 1 min at room temperature. Then fluorescently labeled amino acids were analyzed by isocratic elution on a 4.6 mm i.d. × 250 mm, 5 μm, Inertsil ODS-2 column (GL Sciences Inc., Tokyo, Japan) thermalized at 40 °C with 20 mM sodium acetate/methanol (45/55, *v/v*) at a flow rate of 0.7 mL/min. The fluorescence at 360 nm/460 nm was detected.

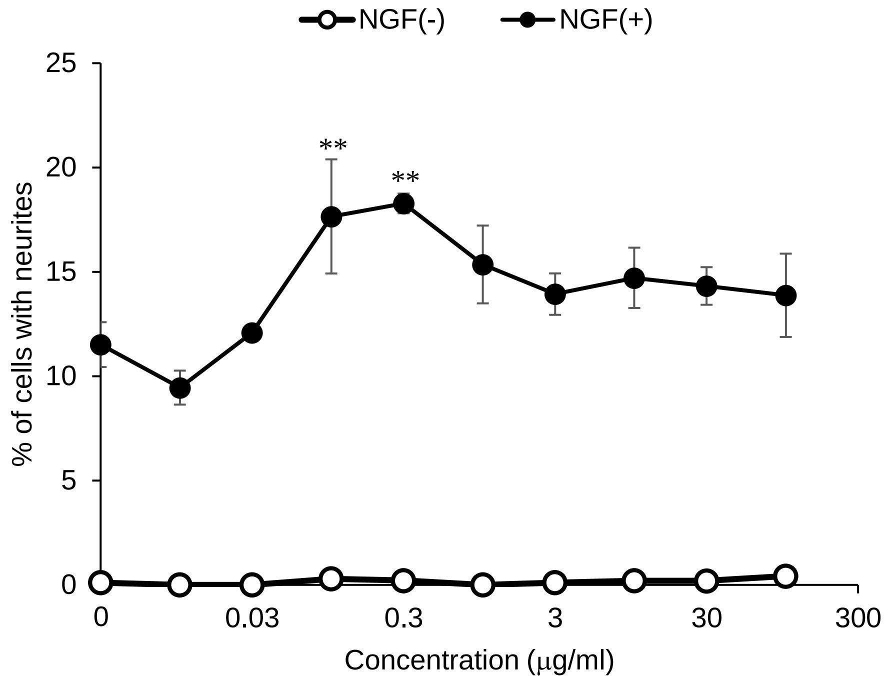

Figure S1. Neurite outgrowth activity of cockscomb hydrolysate in the presence or in the absence of NGF. PC12 cells were plated at 2.0 × 10^3 cells/well and cultured with the samples at 0.01-100 μg/mL in the absence of NGF or in the presence of 10 ng/mL of NGF. The extent of neurite outgrowth was measured at 48 h and is expressed as the mean percentage of 300-400 cells. The data represent means ± standard deviation from triplicated cultures. $^{**}p < 0.01$ (Dunnett's test) as compared with the control (10 ng/mL of NGF).

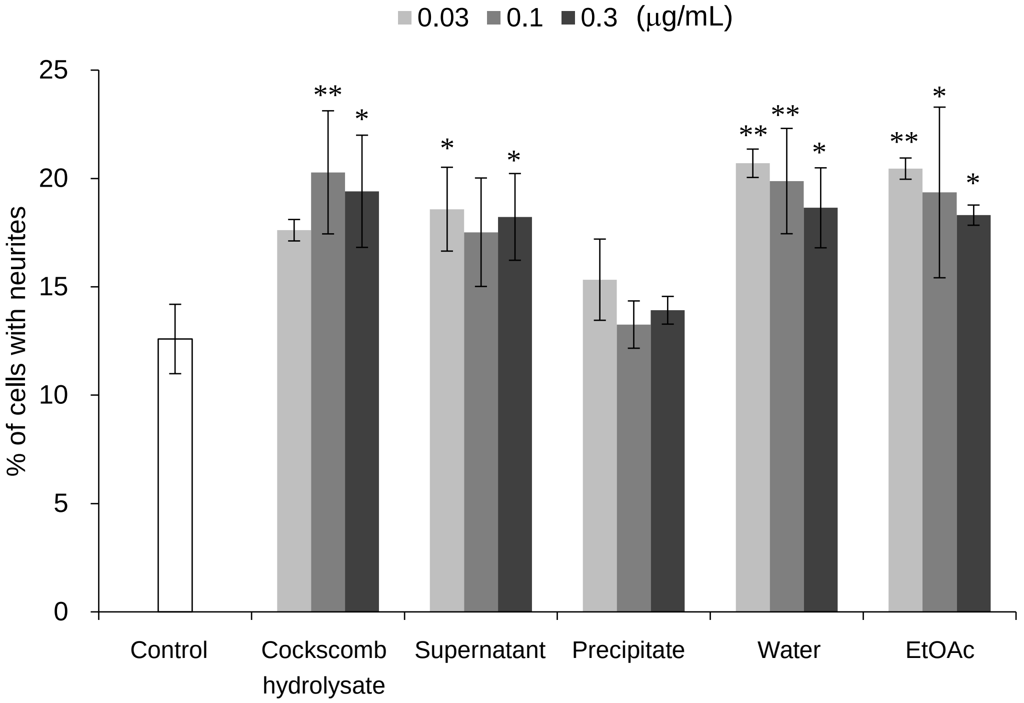

Figure S2. Neurite outgrowth-promoting activities of cockscomb hydrolysate, supernatant, and precipitate by ethanol precipitation, water layer, and EtOAc layer obtained by liquid separation of the supernatant in the presence of Bt_2cAMP. PC12 cells were plated at 4.0×10^3 cells/well and cultured with the samples at 0.03, 0.1 and 0.3 μg/mL in the presence of 0.5 mM Bt_2cAMP. The extent of neurite outgrowth was measured at 24 h and is expressed as the mean percentage of 300-400 cells. The data represent means ± standard deviation from triplicate cultures. $^{*}p < 0.05$, $^{**}p < 0.01$ (Dunnett's test) as compared with the control (0.5 mM of Bt_2cAMP).

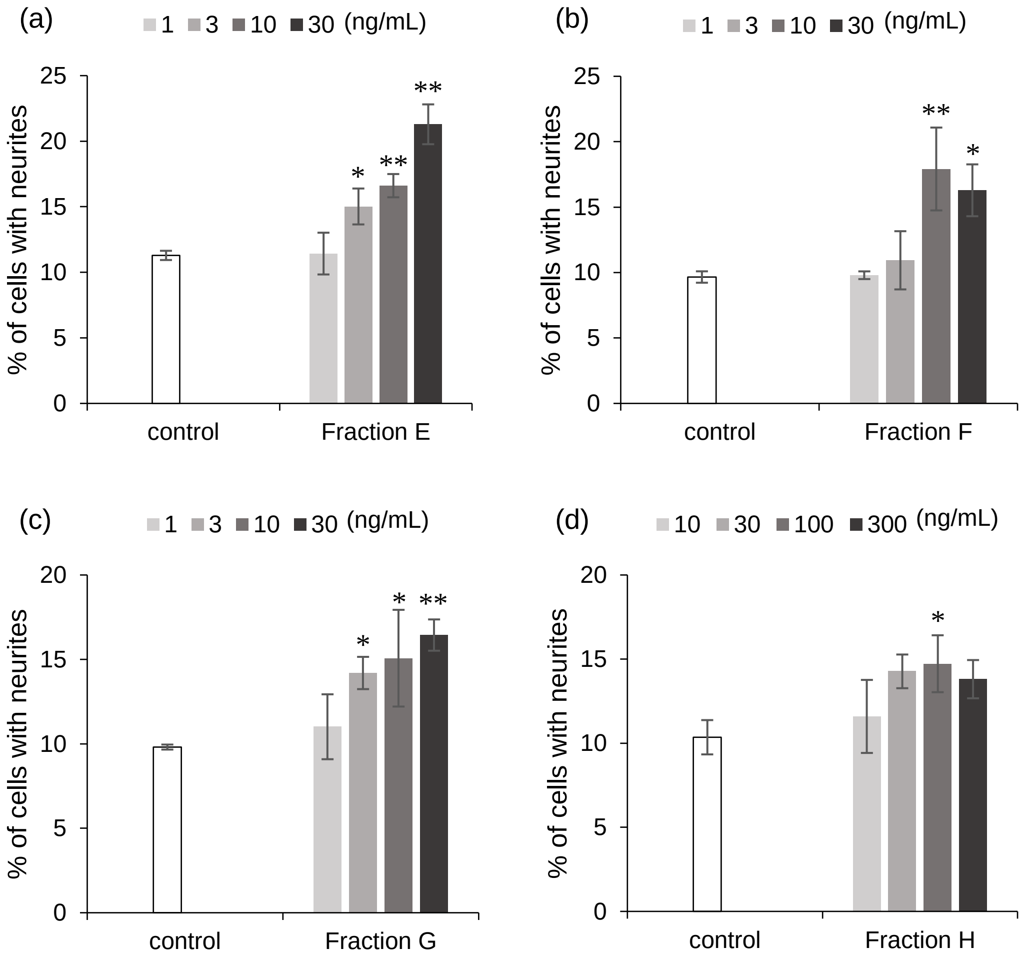

Figure S3. Neurite outgrowth-promoting activities of fractions E-H in the presence of Bt_2cAMP. PC12 cells were plated at 4.0×10^3 cells/well and cultured with the fraction E (a), fraction F (b), fraction G (c), or fraction H (d) in the presence of 0.5 mM Bt_2cAMP. The extent of neurite outgrowth was measured at 24 h and is expressed as the mean percentage of 300-400 cells. The data represent means ± standard deviation from triplicate cultures. $^{*}p < 0.05$, $^{**}p < 0.01$ (Dunnett's test) as compared with the control (0.5 mM of Bt_2cAMP).

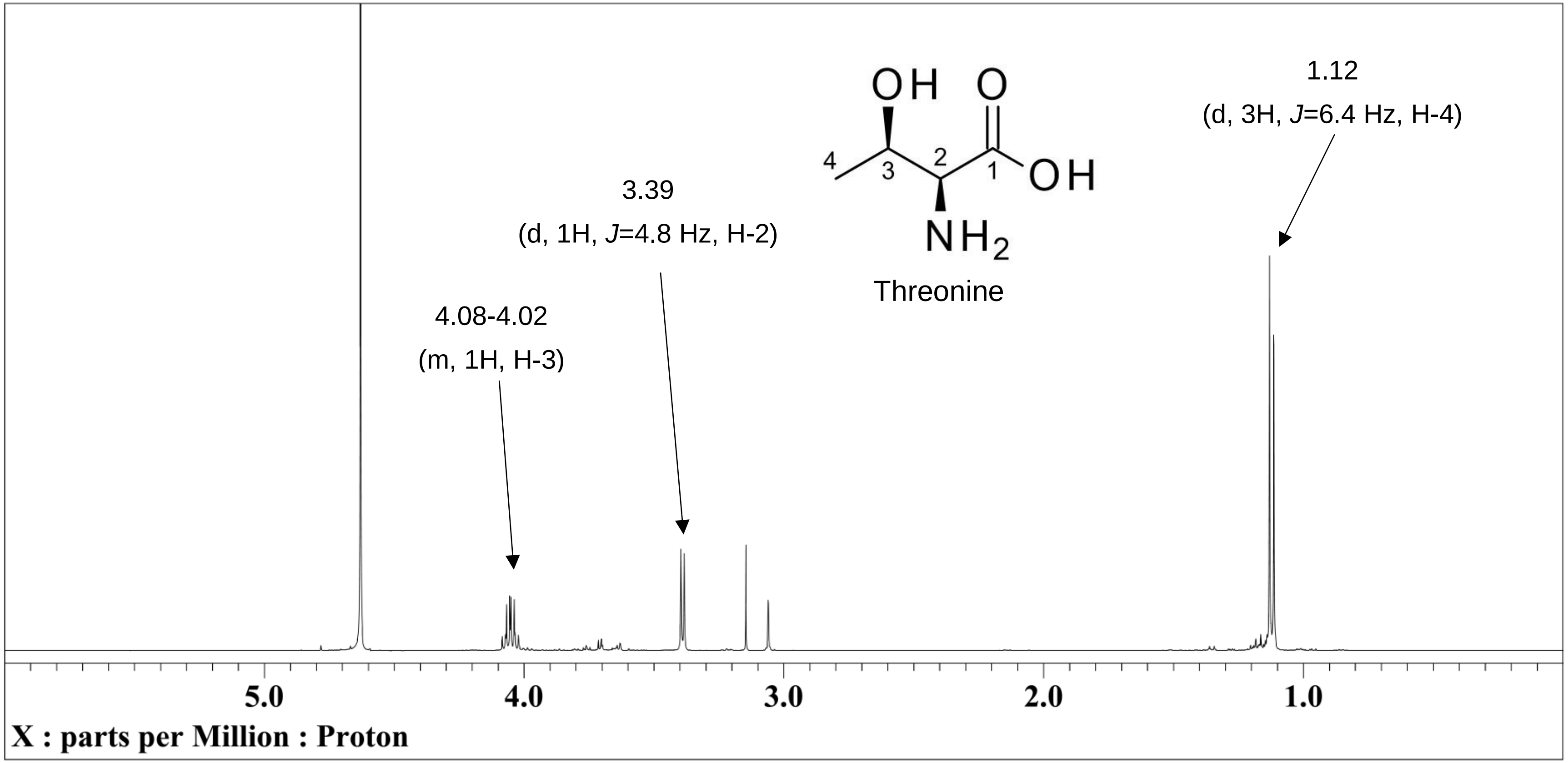

Figure S4. ^{1}H-NMR spectrum of fraction E.

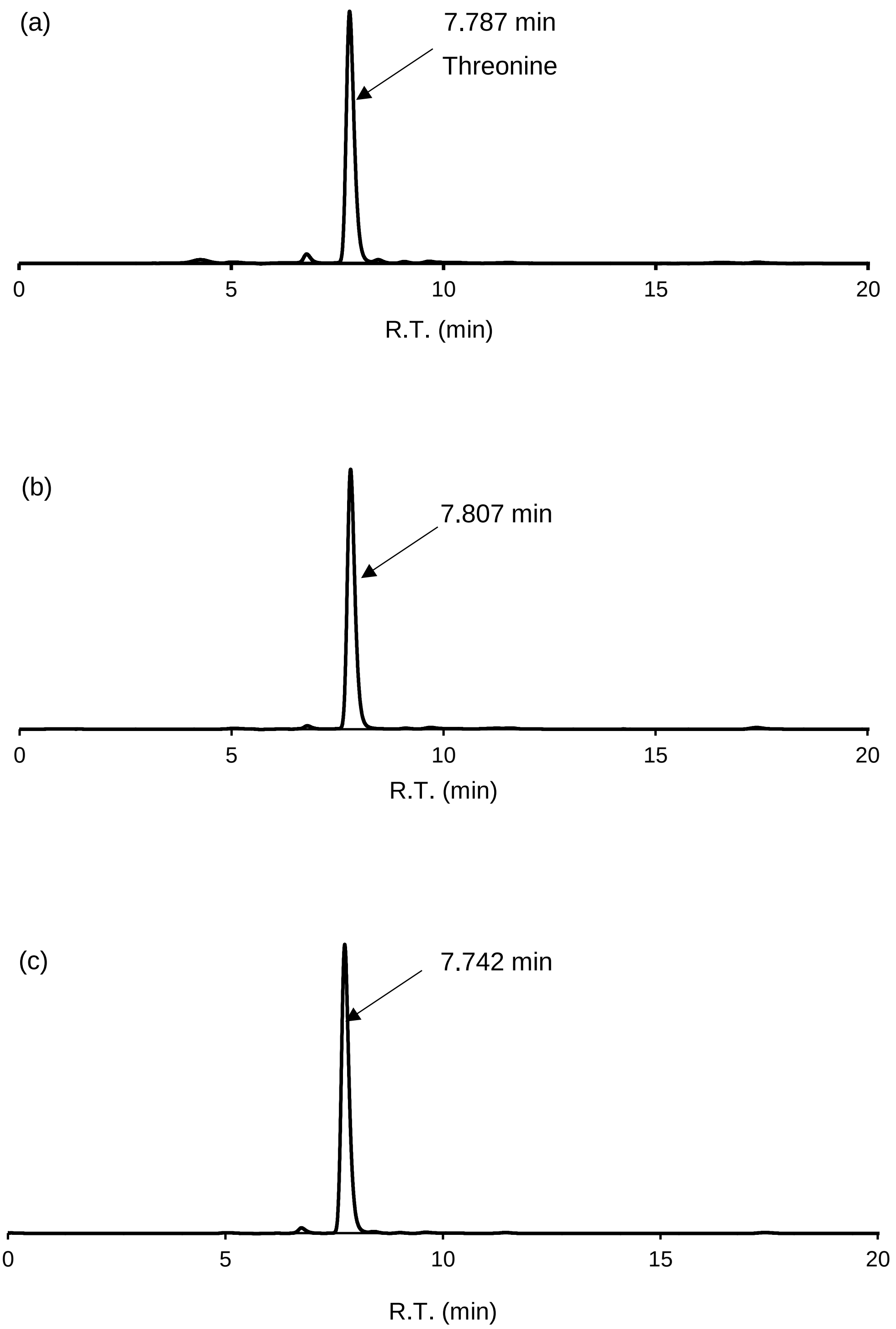

Figure S5. HPLC analyses of fraction E. (a) HPLC analysis of fraction E. (b) HPLC analysis of standard of threonine. (c) HPLC co-chromatography analysis of fraction E and threonine of standard.

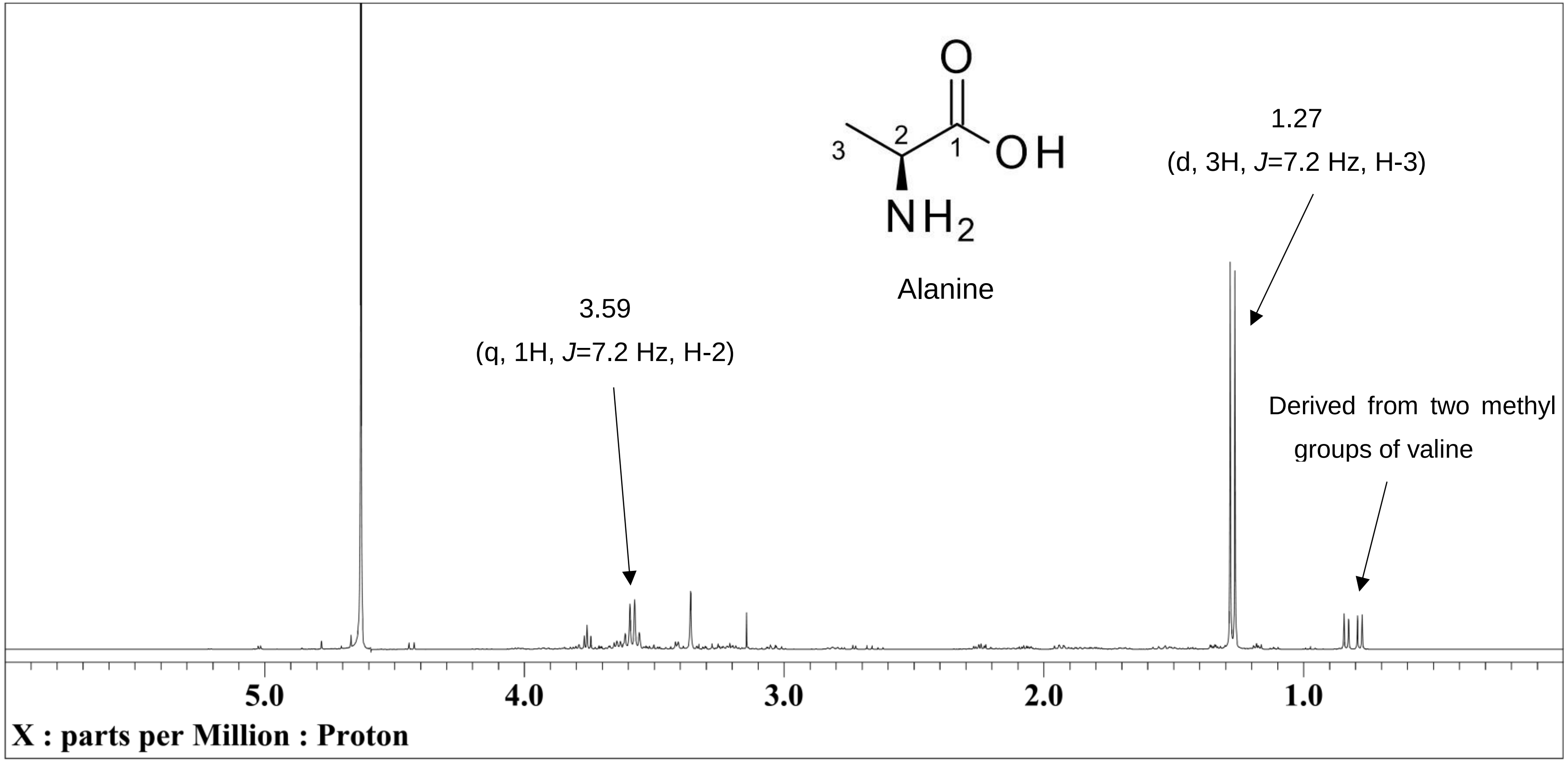

Figure S6. ^{1}H-NMR spectrum of fraction F.

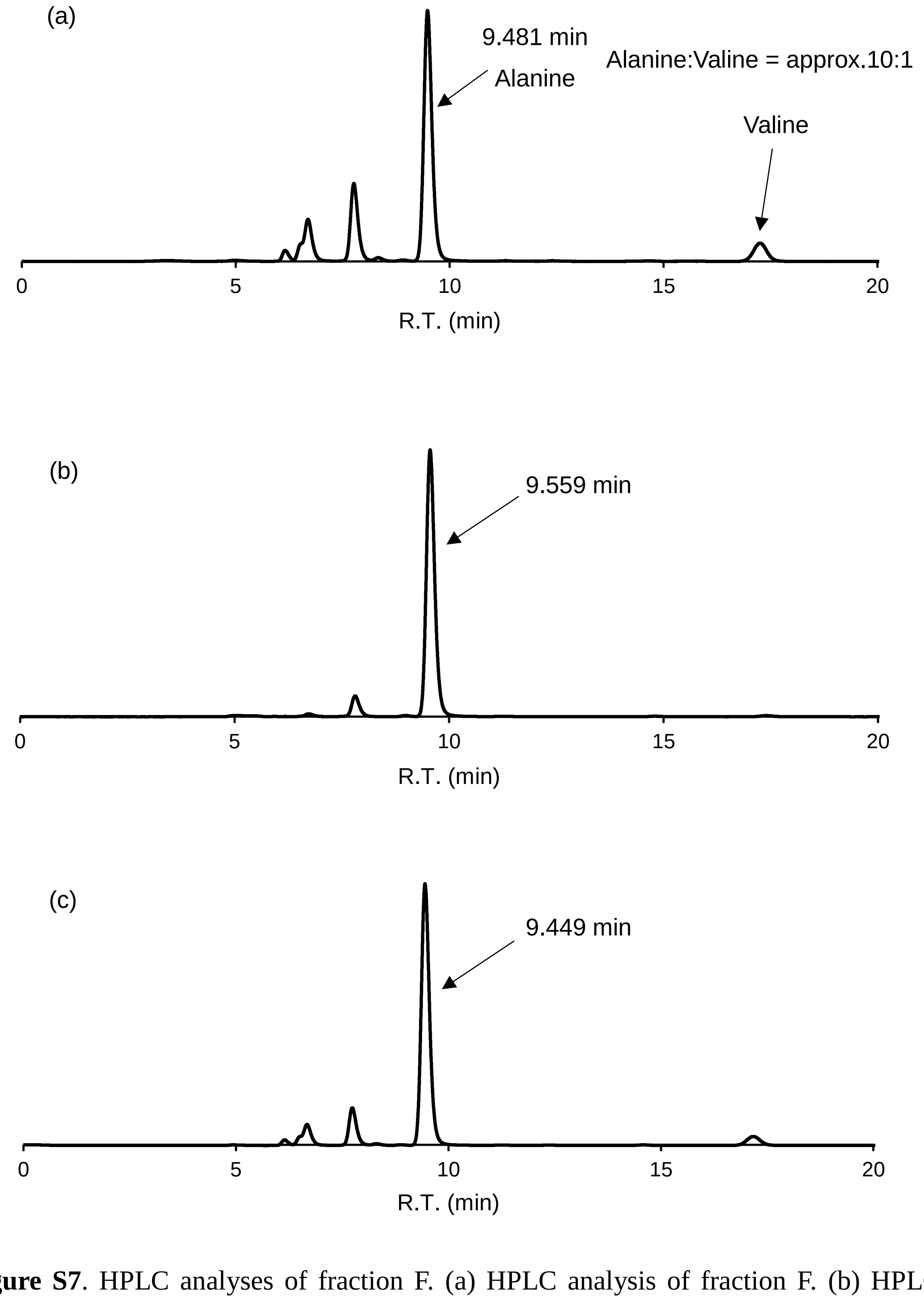

Figure S7. HPLC analyses of fraction F. (a) HPLC analysis of fraction F. (b) HPLC analysis of alanine of standard. (c) HPLC co-chromatography analysis of fraction F and alanine of standard.

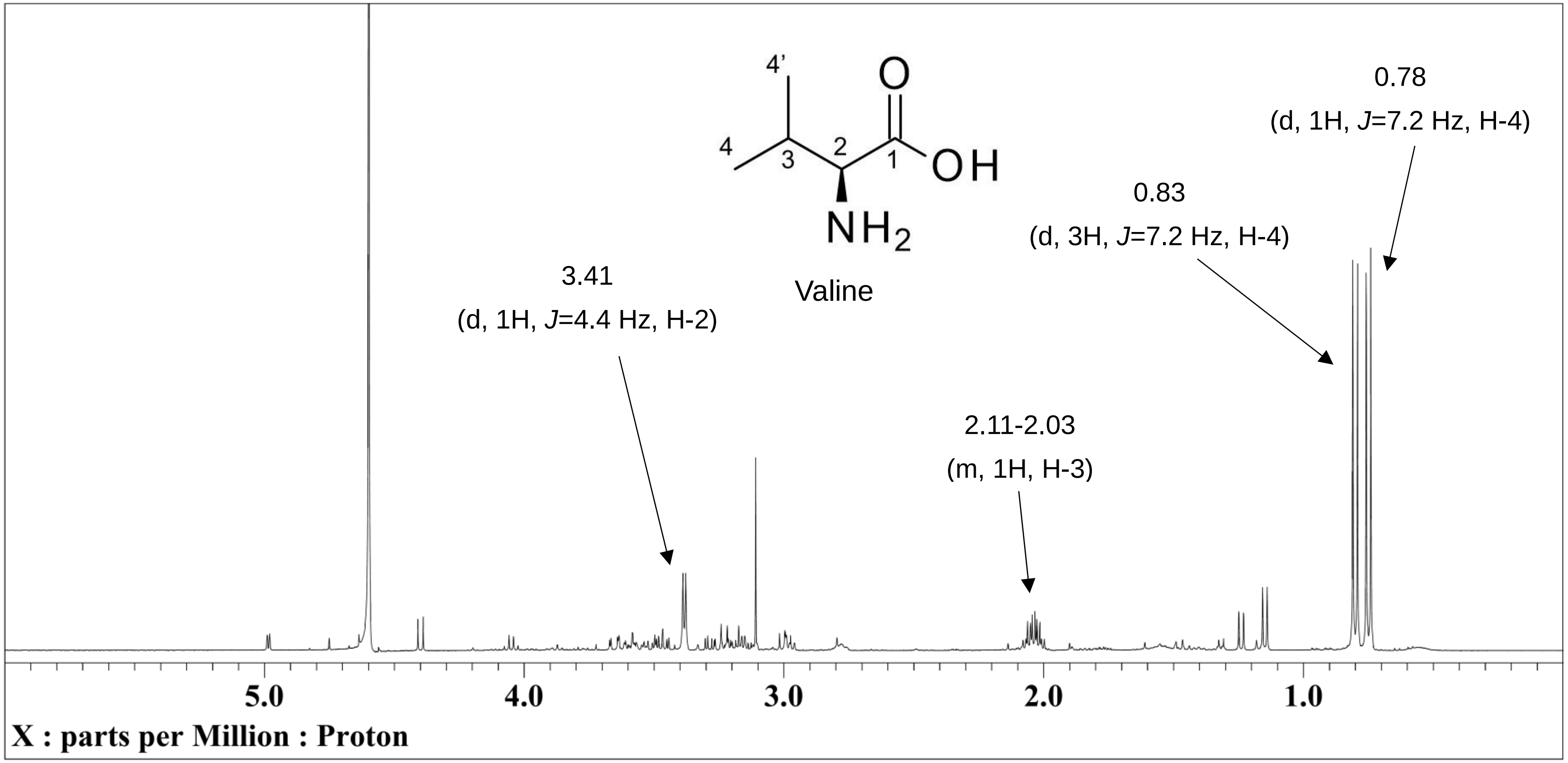

Figure S8. ^{1}H-NMR spectrum of fraction G.

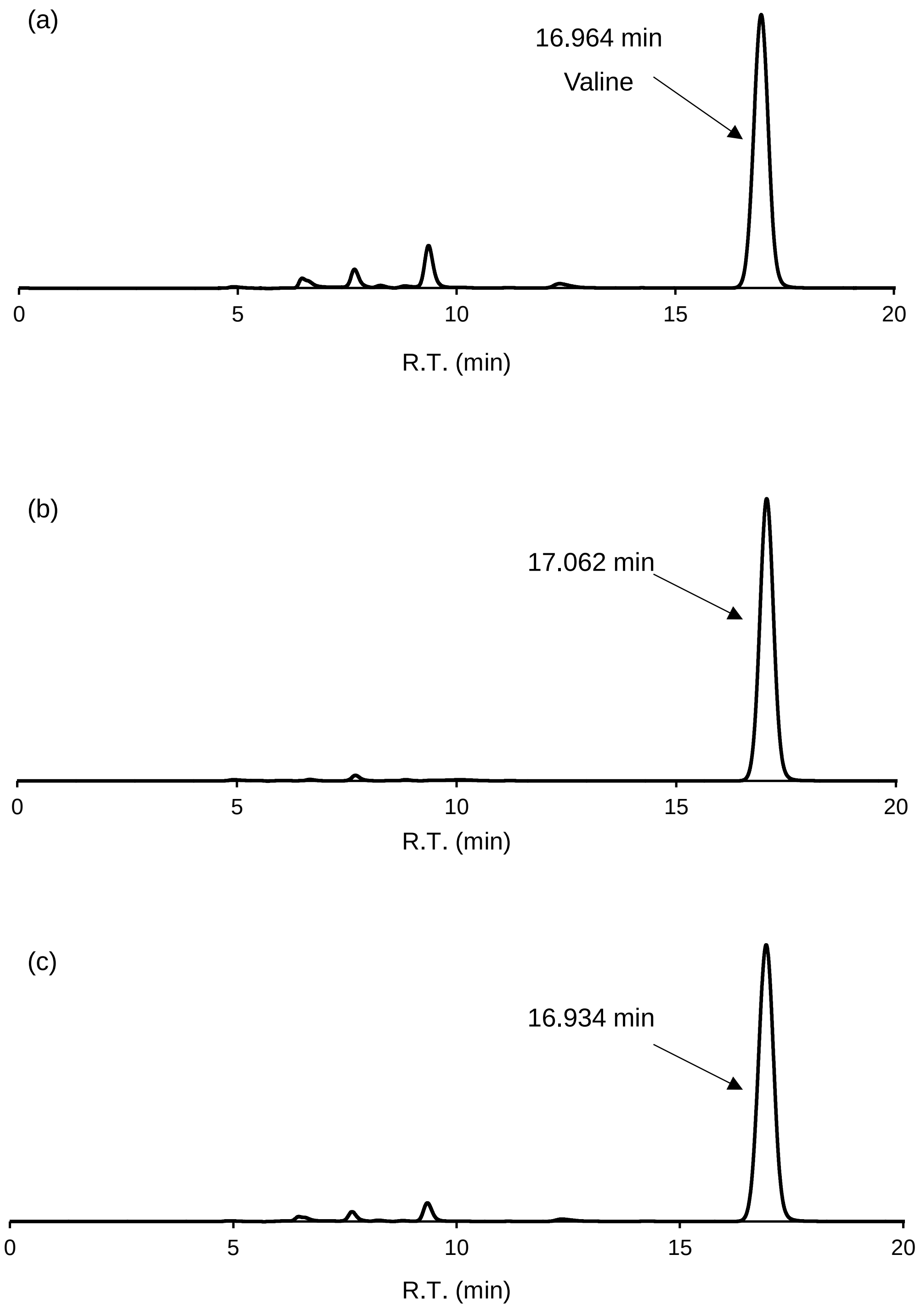

Figure S9. HPLC analyses of fraction G. (a) HPLC analysis of fraction G. (b) HPLC analysis of valine of standard. (c) HPLC co-chromatography analysis of fraction G and valine of standard.

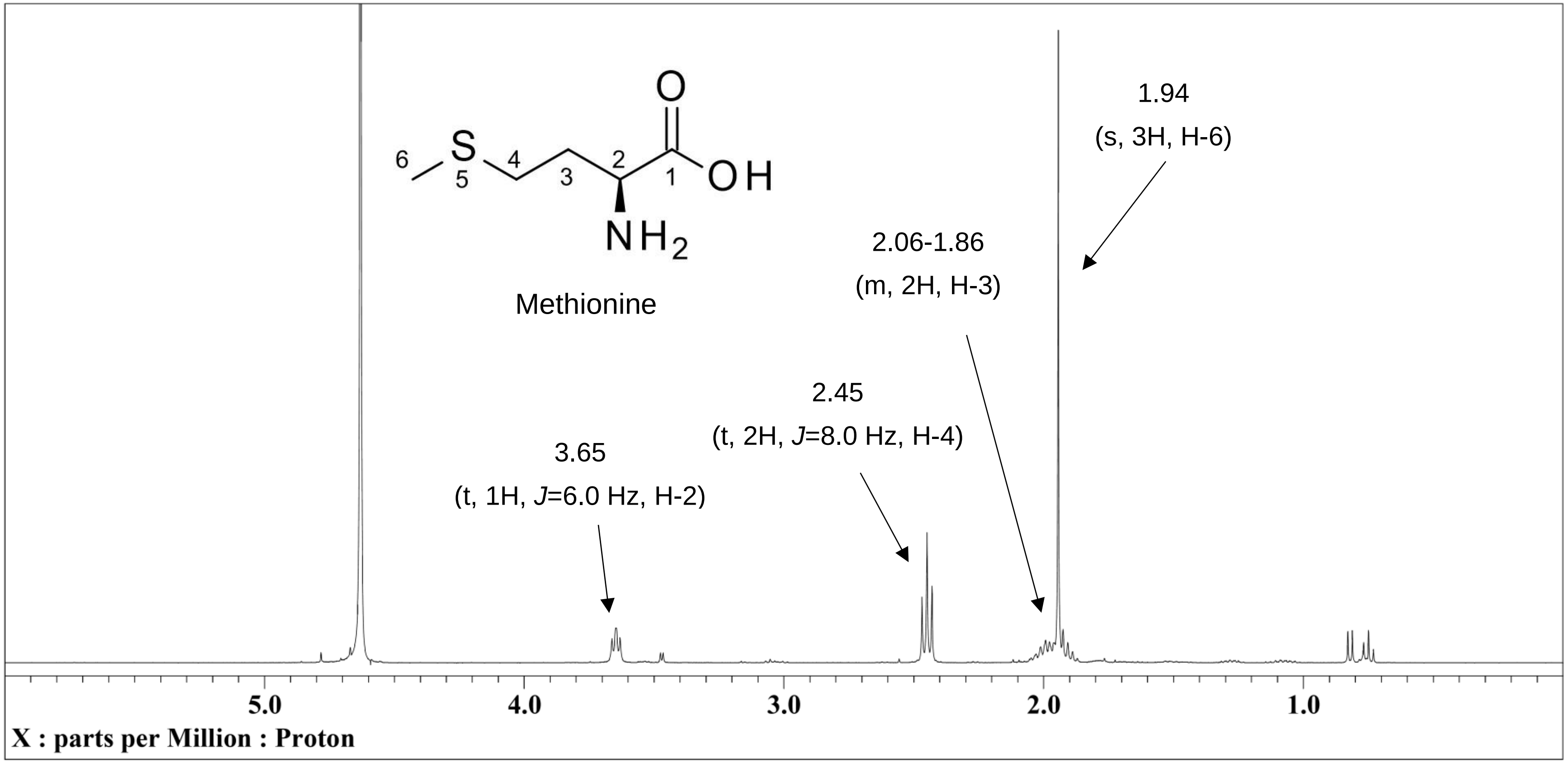

Figure S10. ^{1}H-NMR spectrum of fraction H.

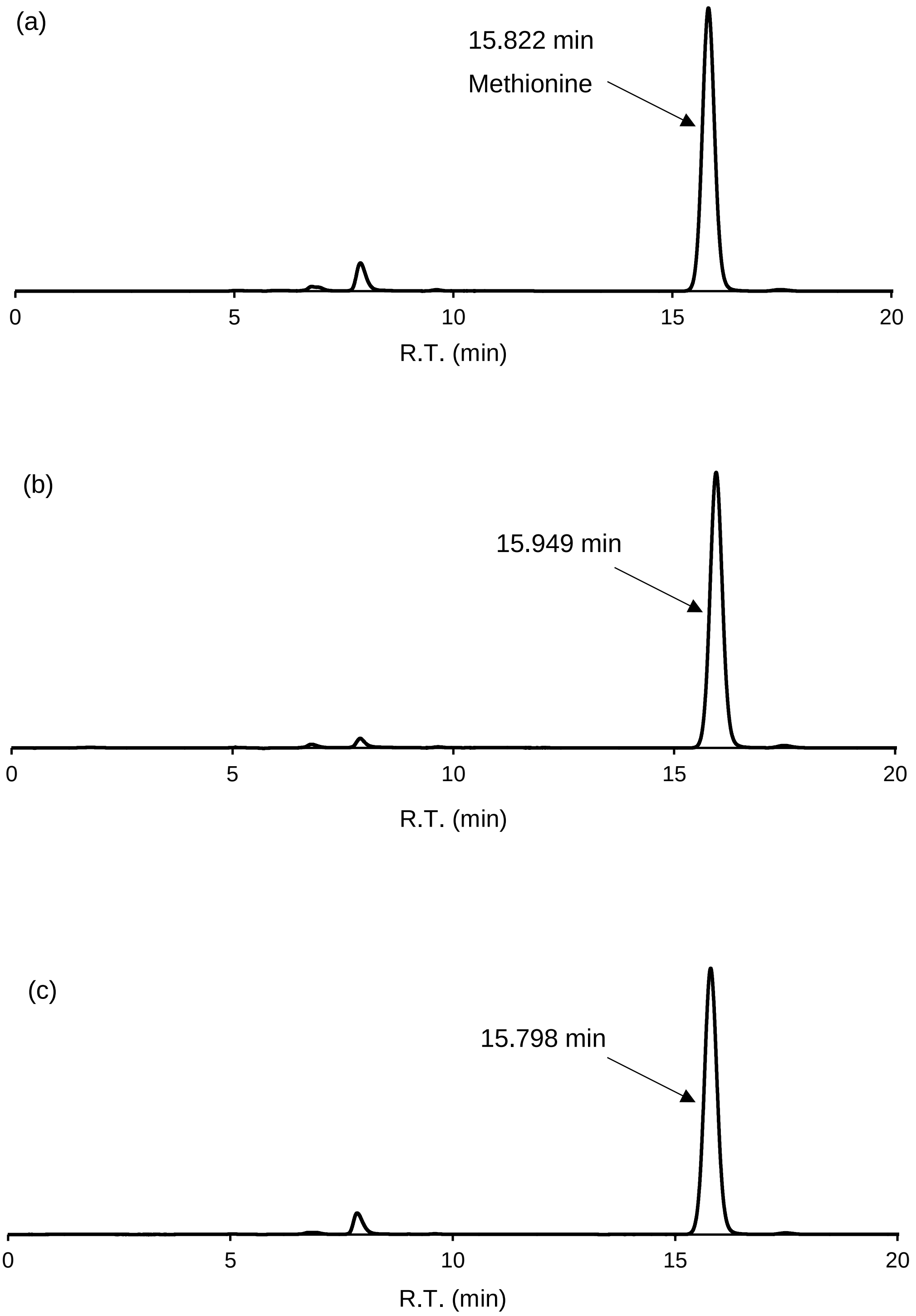

Figure S11. HPLC analyses of fraction H. (a) HPLC analysis of fraction H. (b) HPLC analysis of methionine of standard. (c) HPLC co-chromatography analysis of fraction H and methionine of standard.

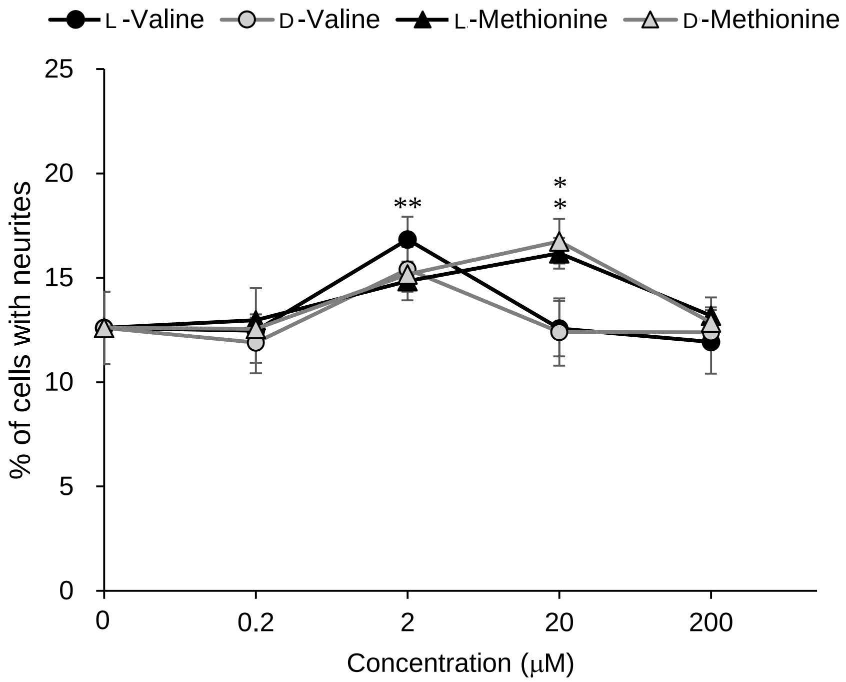

Figure S12. Neurite outgrowth-promoting activities of L-valine, D-valine, L-methionine, and D-methionine in the presence of Bt_2cAMP in PC12 cells. PC12 cells were plated at 4.0×10^3 cells/well and cultured with the amino acids at 0.2-200 μM in the presence of 0.5 mM of Bt_2cAMP. The extent of neurite outgrowth was measured at 24 h and is expressed as the mean percentage of 300-400 cells. The data represent means ± standard deviation from three independent experiments. $^{*}p < 0.05$, $^{**}p < 0.01$ (Dunnett's test) as compared with the control (0.5 mM Bt_2cAMP only).

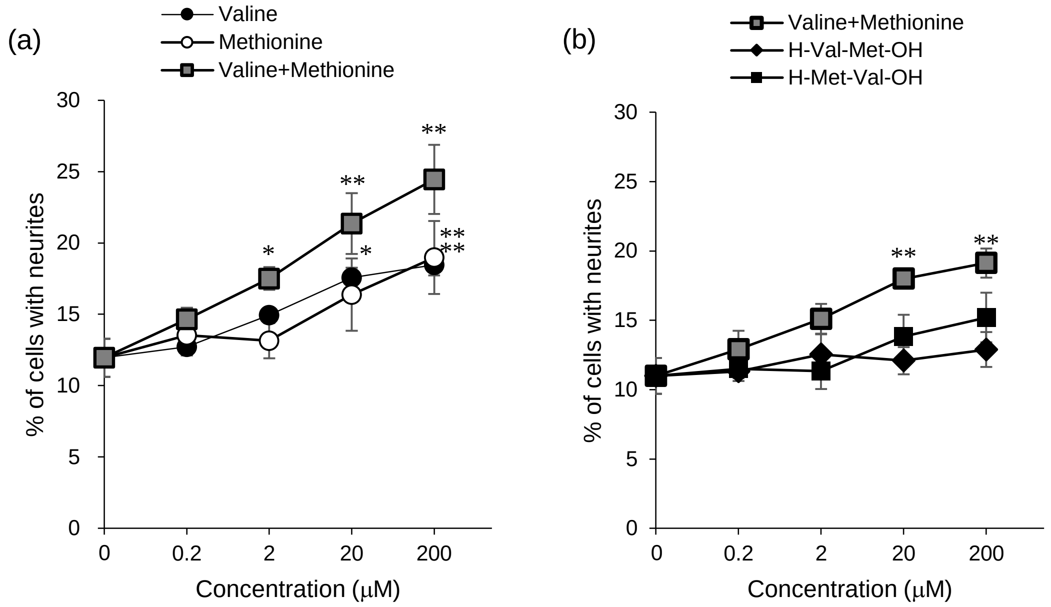

Figure S13. Neurite outgrowth-promoting activities of valine, methionine, their mixture, and their dipeptides in the presence of NGF in PC12 cells. (a) Promoting activities of valine and methionine and a mixture of equal amounts of valine and methionine for neurite formation induced by NGF in PC12 cells. (b) Promoting activities of a mixture of equal amounts of valine and methionine and their dipeptides (H-Val-Met-OH, H-Met-Val-OH) for neurite formation induced by NGF in PC12 cells. PC12 cells were plated at 2.0×10^3 cells/well and cultured with the samples at concentration of 0.2-200 μM in the presence of 10 ng/mL of NGF. The extent of neurite outgrowth was measured at 48 h and is expressed as the mean percentage of 300-400 cells. The data represent means ± standard deviation from three independent experiments. $^{*}p < 0.05$, $^{**}p < 0.01$ (Dunnett's test) as compared with the control (10 ng/mL of NGF only).

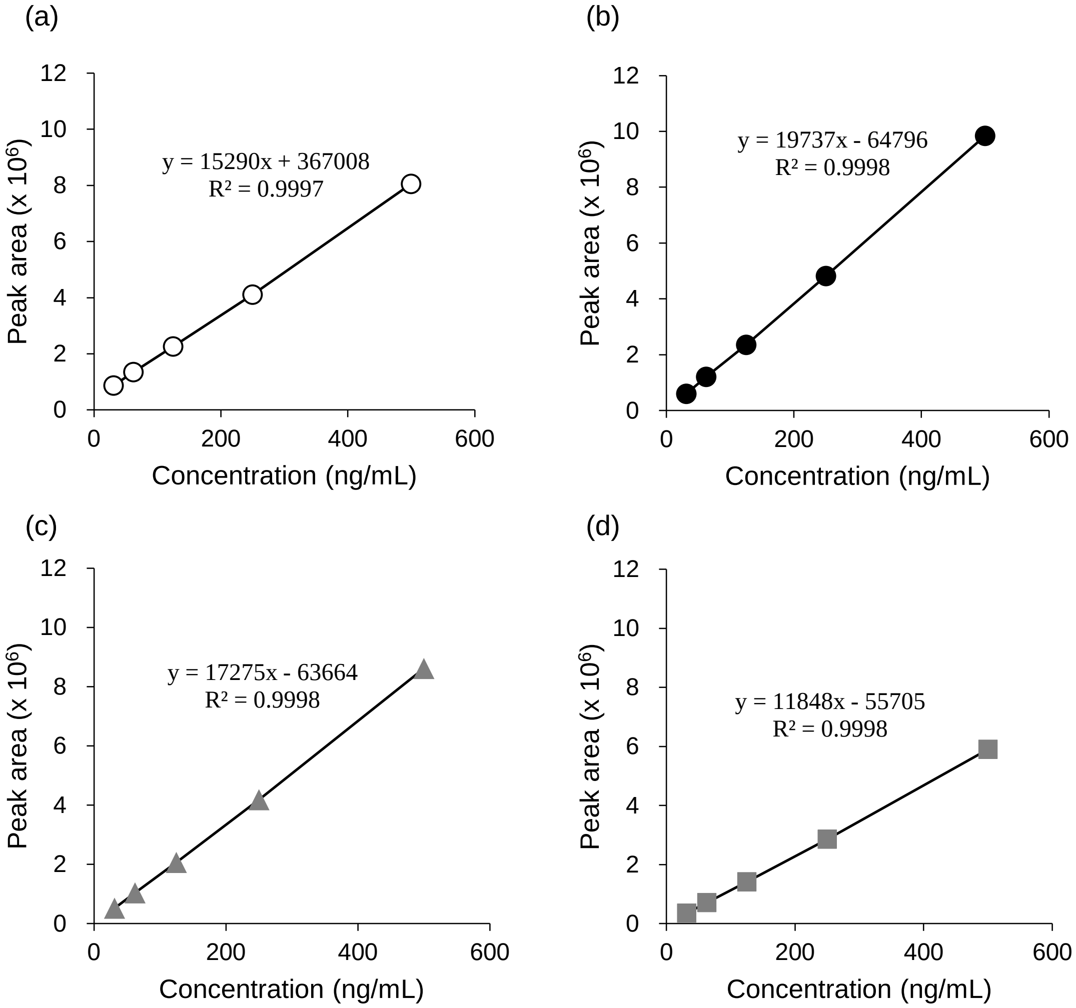

Figure S14. Calibration curves of the reference standards of threonine, alanine, valine, and methionine. Calibration curves of threonine (a), alanine (b), valine (c), and methionine (d) were prepared by plotting the concentration of each compound vs. the area of the peak. Amino acids were labeled with OPA and detected the fluorescence at 360 nm/460 nm.

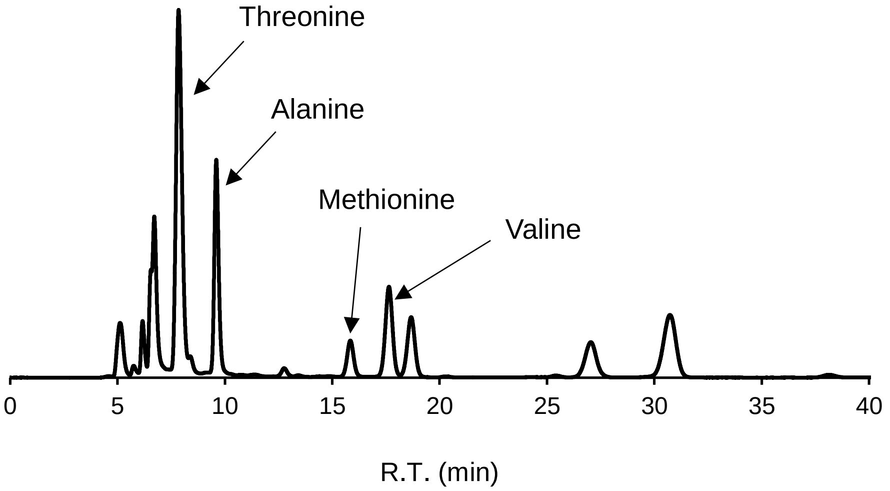

Figure S15. HPLC analysis of the water layer of cockscomb hydrolysate.

Table S1. Quantification of threonine, alanine, valine, and methionine in the water layer (5 μg/mL) of cockscomb hydrolysate.

Cockscomb hydrolysate (5 μg/mL)			
samples	R.T. (min)	peak area	concentration (ng/mL)
Threonine	7.843	4889224	343.8
Alanine	9.598	2218838	115.7
Methionine	15.842	518548	48.5
Valine	17.641	1435259	86.8

Concentration of each compound was determined from the relative peak area with reference to a calibration plot (Figure S14) obtained with the authentic commercial standard.
